# Renal Hemosiderosis among Iranian Transfusion Dependent β-Thalassemia Major Patients

**Published:** 2017-04-01

**Authors:** Mozhgan Hashemieh, Mitra Radfar, Azita Azarkeivan, Seyed Mohammad Taghi Hosseini Tabatabaei, Sedigheh Nikbakht, Mehdi Yaseri, Kourosh Sheibani

**Affiliations:** 1 Imam Hossein Medical Center, Shahid Beheshti University of Medical Sciences, Tehran, Iran; 2 Research Center of Iranian Blood Transfusion Organization, Thalassemia Clinic, Tehran, Iran; 3 Imam Hossein Medical Center, Department of Pediatric Nephrology, Shahid Beheshti University of Medical Sciences, Tehran, Iran; 4 Pediatrition, Yasuj, Iran; 5 School of Public Health and Public Health Research Institute, Tehran University of Medical Sciences, Tehran, Iran; 6 Basir Eye Health Center, Basir Health Clinic, Tehran, Iran

**Keywords:** β-Thalassemia major, Renal involvement, Transfusion, MRI T2*, Iran

## Abstract

**Background: **In recent years, the success in management of thalassemic patients, has allowed for some previously unrecognized complications including renal abnormalities to emerge. This prospective study aimed to investigate kidney iron overload by means of MRI T2* and also renal function based on laboratory tests for early markers of glomerular and tubular dysfunction among adult Iranian transfusion-dependent thalassemia major patients.

**Subjects and Methods:** Two-hundred and two patients with transfusion-dependent β-thalassemia major were included in this study in Zafar Adult Thalassemia Center, Tehran, Iran. For all patients, kidney MRI T2* as well as evaluation of BUN, creatinine, uric acid, calcium, phosphorus, sodium (Na), potassium (K), total protein, albumin, cystatin C, serum ferritin β2-microglobulin, NAG (N-acetyl-beta-D-Glucosaminidase), and urine protein were performed.

**Results: **One-hundred and fourteen female and 88 male transfusion-dependent β-thalassemia major patients with mean age of 30.1 ± 9.4 participated in the present study. We found that 77.7% of our patients had kidney hemosiderosis based on MRI T2*. Also, 67 patients (33.2%) had elevation of serum cystatin C, and 104 patients (51.5%) had reduced estimated glomerular filtration rate (e-GFR). Increased urinary excretion of NAG and hypercalciuria were found in 50% and 79.2% of participants, respectively.

**Conclusion:** Renal hemosiderosis and asymptomatic renal dysfunction are prevalent among transfusion- dependent β-thalassemia major patients which necessitate regular screening with early markers of glomerular and tubular dysfunction. Further studies in order to investigate the correlation between renal hemosiderosis and early markers of kidney dysfunction among these patients are recommended.

## Introduction

 Thalassemia syndromes are the most common single gene disorders worldwide, presenting a significant public health problem for developing countries.^1^In β-thalassemia major (β-TM), both β globingenes are mutated, and the production of β globin chains is impaired, resulting in severeanemia.^2^ In patients with thalassemia Intermedia (TI), the production of β globin chains is decreased and the clinical severity of thalassemia ranges between the mildsymptoms of thalassemia trait and the severe symptoms of thalassemia major.^2^Most patients with β-TM have profound anemia necessitating regular blood transfusion to survive. Repeated blood transfusions are inevitably associated with iron accumulation in different vital organs.^3^

 The success in management of β-TM patients has led to chronic hemosiderosis in different organs like liver and heart due to prolonged transfusions.^4^Also, the importance of the long-term complications due to hemosiderosis in other organs like pancreas and kidneys have recently been studied.^5^, ^6^

The evidence of proximal tubular damage has been observed in β-TM patients.^3^,^7^ Also, low-molecular-weight proteinuria has been found in almost all patients.^8^Moreover, several studies have reported increased urinary excretion of markers for proximal tubular damage in patients with β-TM, including N-acetyl-b-D-glucosaminidase and β 2-microglobulin, calcium, phosphate and magnesium, uricacid, aminoacids, and malondialdehyde.^7^-^9^In another study, authors have reported renal hyperfiltration, hypercalciuria and albuminuria to be common in transfusion-dependentthalassemia.^10^

Beta-thalassemia major patients also show significantly higher levels of cystatin C as a marker of glomerulardysfunction.^11^Cystatin C has been suggested as a sensitive marker of glomerular filtration rate (GFR) providing an early indication of renal impairment, possibly superior to serumcreatinine.^12^Cystatin C and serum β2 microglobulin show a strong correlation with creatinine clearance and age, while NAG positivelycorrelateswithproteinuria.^13^

Chronicanemia, iron overload and the use of specific iron chelators have an important role in the pathogenesis of kidney dysfunction among patients with β-TM.^8^ A relation between chronic anemia and hypoxia with oxidative stress and lipid peroxidation has been documented and lipid peroxidation can cause functional abnormalities in tubularcells.^14^Anemiacancan lead to tubuloin terstitial hypoxia and this phenomena causes apoptosis or epithelial-mesenchymal transition, leading to the development of tubulointerstitial injury and consequent glomerulosclerosis and kidneyfibrosis.^15^ These changes in long term can lead to a decline in GFR among thalassemic patients.^3^

Extensive intramedullary destruction of red cell precursors, shortened RBC survival, rapid iron turnover and regular blood transfusion lead to iron accumulation in patients with β-TM.^16^ Iron accumulation in the heart and liver is a well known cause of life-threatening complications such as cardio myopathy and cirrhosis, but the significance of iron accumulation in the kidneyispoorlyunderstood.^17^Moreover, in patients with β-TM, the urine markers of tubular dysfunction correlate positively with serum ferritin and liver iron deposition as detected by MRI T2 values.^18^

The present study was conducted to evaluate the glomerular and tubular function in patients with β-TM and also to assess the kidney iron overload by means of MRI T2*among patients with β-TM.

## MTERIALS AND METHODS

Patients

This study was conducted in Zafar Thalassemia Center, a referral center in Tehran, Iran from May to October 2015.Regularlytransfused patients with β-TM were enrolled in this prospective study. Exclusion criteria were: diabetes mellitus, liver disease, renal dysfunction, cardiomyopathy and diuretic therapy. This study was approved by the Ethics Committee of Shahid Beheshti University of Medical Sciences and written consent was obtained from all participants.

Magnetic Resonance Imaging

For all patients, MRI T2* of kidney was performed to asses iron overload. Patients were scanned using a symphony 1.5 Tesla Scanner (Philips, Netherland). A standard RF body coil was used in all measurements as described before.^5^ The Royal Brompton protocol based on a single breath multi-echo fast gradient-echo sequence was used for T2 measurement. The kidney T2* was performed by imaging a single trans-axial slice (10 mm) through the center of the kidney.T2* values were calculated for patients using an in-house software (Pardis Noor Medical Imaging Center, Tehran, Iran).A homogenous region of interest (ROI) derive was outlined in the kidney parenchyma. The mean signal intensity of region was measured for each image, and plotted against the echo time (TE). Iron overload in the kidney was defined by T2* values <65 millisecond.

**Laboratory Tests**

In current study, we measured cystatin C, Na, K, uric acid, calcium, phosphorus, total protein, albumin, urea, creatinine and serum ferritin. Also, we assessed urinary β2-microglobulim, NAG, and specific gravity. Urine creatinine, uric acid, calcium and phosphorus were assayed by photometric method using Cobas Integra 400 plus analyser and a diagnostic kit (Roche, Switzerland). Urine sodium and potassium were measured by Ion Selective Electrode (ISE) method with a Caretium device. Urine protein was evaluated by photometric method with a spectrophotometer device and the specific gravity of urine was measured using a refractometer device. NAG was assessed by Elisa method using Cusabio kit and urine β2 microglobulin was evaluated by nephelometry using a diagnostic kit (Binding site-England). All of these studies were performed on 20 cc of first morning fresh urine sample. Measurement of serum ferritin was carried out using electro chemiluminescence (Elecsys 2010 Chemistry Analyzer, Roche Diagnostic, Basel, Switzerland). Estimated GFR was calculated using Schwartz formula and renal dysfunction was defined as e-GFR<90 ml/min/1.73 m2 (20). Cystatin C was assayed by turbidimetry method and a Mindray BS 400 device using the related diagnostic kit (Gention, Norway).

**Estimation of Sample Size and Statistical Analysis**

In order to have a power of 90% and considering an estimated prevalence of 70% for kidney hemosiderosis (based on a pilot study), total sample size was determined to be 198 subjects. We included 202 samples in our study. To present data, we used mean, standard deviation, median and range, frequency and percent. All statistical analysis was performed using SPSS software version 22 (Armonk, NY: IBM Corp).

## Results

 Between May and October 2015, 202 patients (114 females, 88 males) were included in the present study. The mean age was 30.1 ± 9.4 with a range of10-68 years. The mean age of patients at the beginning of transfusion was 10 ± 2 months. Demographic characteristics of patients are shown in Table 1.

**Table 1:** Demographic characteristics of patients entering the study

		Total
	
Age	Mean ± SD	30.1 ± 9.4
	Median (range)	30 (10 to 68)
Gender	Female	114 (56.4%)
	Male	88 (43.6%)
Desferal Per week	Mean ± SD	15.4 ± 15.1
	Median (range)	14 (0 to 210)
Age at Transfusion	Mean ± SD	10 ± 2
	Median (range)	9 (2 to 19)
Age at Desferal	Mean ± SD	35.2 ± 44.5
	Median (range)	24 (0 to 564)
Height(cm)	Mean ± SD	163 ± 10
	Median (range)	162 (120 to 188)
Weight(kg)	Mean ± SD	56.2 ± 10
	Median (range)	55 (22 to 90)
BMI	Mean ± SD	21.2 ± 3.6
	Median (range)	20.8 (11.5 to 35.6)

*Based on Chi-square test.

† Based on t-test.

‡ Based on Mann-Whitney test.

The mean value of serum ferritin was 1660 ± 1458 ng/ml and 117 patients (57.9%) had ferritin level more than 1000 ng/ml. Also, the mean usage of desferrioxamine vials per week was 15.4 ± 15.1. The mean age at thestart of iron chelation therapy with desferrioxamine was 35.2 ± 44.5 months (Table 1). In this study, the mean relaxation time for kidney MRI T2* was 50 ± 17 millisecond. Kidney Iron overload was defined by T2* values <65millisecond. Based on this threshold, 157 patients (77.7%) had kidney hemosiderosis. The mean e -GFR in our patients was estimated to be 129.9 ± 50.8 cc/minute. Based on our findings, e -GFR was below the normal range in 104 patients (51.5%) according to their age and sex. Also, elevation of serum cystatin C and urine NAG was observed in 33.2% and 50% of patients, respectively and the ferritin level in 117 patients was more than 1000 ng/ml. In our study, only 5% of patients had elevation of β2-microglobuline in serum (Table 3) and the urine calcium to creatinine ratio was elevated in 160 patients (79.2%)(Tables 2,3).

**Table 2:** Paraclinical findings among patients entering the study

	**Mean**	**SD**	**SE**	**Median**	**Min**	**Max**
MRI T2* ( Relaxation time )	50	17	1	49	8	85
BUN	31.3	16	1.1	26	14	98
GFR(cc/min)	129.9	50.8	3.6	123.6	25.6	286
Serum Creatinine	0.69	0.3	0.02	0.6	0.3	2.6
Serum Uric Acid	5.5	1.5	0.1	5.3	2.5	13.2
Serum Calcium	9.1	0.8	0.1	9.1	6	11
Serum phosphor	4.95	1.05	0.07	4.85	2.7	9
Serum Na	136.5	3	0.2	136	129	144
Serum k	4.8	0.5	0	4.7	3.4	6.9
Serum total protein	7	0.8	0.1	7	5.1	9.2
Serum Albumin	4.5	0.5	0	4.5	3.4	5.9
Serum Cystatin C	6.2	74.8	5.3	0.9	0.1	1064
Urine NAG	107	265	19	15	5	2000
Serum Ferritin	1660	1458	103	1200	0	13110
Urineβ2MICRO	0.24	0.99	0.07	0.1	0.03	8.9
Urine Specific Gravity	1.02	0.02	0	1.02	1.01	1.25
Urine Creatinine	87.4	41.7	2.9	84	17	274
Urine Calcium	11.5	10.1	0.7	8.9	0.1	54
Urine Ca/Cr	0.15	0.15	0.01	0.1	0	0.85
Urine Uric acid	42.6	20.1	1.4	41	9	132
Urine Uric acid/Cr	0.56	0.32	0.02	0.49	0.03	1.89
Urine Na	75.4	45.3	3.2	65	10	300
Urine P	43.3	28.8	2	37	6	163
Urine Protein	6	8	1	5	2	63
Urine Protein /Cr	0.11	0.24	0.02	0.06	0.01	2.92

**Table 3:** The frequency and percentage of abnormal findings in patients entering the study

	Abnormal	%	95% CI*	
			Lower	Upper
GFR(cc/min)	104	51.5%	44.5%	58.4%
Kidney MRI T2*	157	77.7%	71.9%	83.5%
Serum BUN	26	12.9%	8.2%	17.5%
Cr	4	2.0%	0.0%	3.9%
Serum uric acid	15	7.4%	3.8%	11.1%
Serum calcium	19	9.4%	5.3%	13.5%
Serum Phosphor	85	42.1%	35.2%	48.9%
Serum Na	0	0.0%	0.0%	0.0%
Serum k	25	12.4%	7.8%	17.0%
Serum total protein	18	8.9%	4.9%	12.9%
Serum Albumin	30	14.9%	9.9%	19.8%
Serum Cystatin C	67	33.2%	26.6%	39.7%
Urine NAG	101	50.0%	43.0%	57.0%
Serum Ferritin	117	57.9%	51.1%	64.8%
Serum β 2MICRO	10	5.0%	1.9%	8.0%
Urine specific gravity	22	10.9%	6.6%	15.2%
Urine ca/cr	160	79.2%	73.6%	84.9%
Urine uric acid/cr	20	9.9%	5.7%	14.1%
Urine protein/cr	9	4.5%	1.6%	7.4%

## Discussion

 Improved survival among thalassemic patients in recent years has led to the manifestation of morbidities such as renal dysfunction.^5^ Since about 20,000 β- thalassemia major patients are covered by the Iranian association of thalassemia patients and considering the high cost of treatment for these patients, it appears necessary and cost-effective to diagnose the renal dysfunction in early stages to prevent ongoing renal injury.^21^ It has been indicated that renal dysfunction in thalassemia increases with age and duration of blood transfusions.^22^ Also, tubulopathy and glomerular dysfunction, as well as hyperfiltration in patients with TM have been reported.^23^^, ^^24^

The underlying mechanism for renal dysfunctions in patients with β-TM seems to be multifactorial, attributed mainly to long-standing anemia, chronic hypoxia, iron overload and deferoxamine toxicity.^25^

In the present study, we found that 77.7% of our patients had kidney iron overload. Many authors have claimed that one of the most important factors in pathogenesis of tubulopathy and glomerular dysfunction is iron accumulation in kidney.^3^^, ^^8^^-^^10^^, ^^24^

Previous studies on kidney MRI T2* in patients with thalassemia are very limited in literature. Koliakos et al. in a study on 91 β-TM patients showed that the urine concentration of albumin, β2 microglobulin and NAG activity correlated positively with serum ferritin and liver iron deposition as detected by MRI T2* values. Based on these findings, they suggested that the cause of renal dysfunction in β-TM is iron overload.^18^

In another study, authors found a moderate correlation between kidney MRI T2* relaxation time and serum ferritin, and a weak correlation between liver and heart T2* relaxation times.^5^

In the present study, 51.5% of patients had reduced levels of e-GFR. This result is in agreement with the findings of similar studies.^22^^, ^^23^^, ^^25^

For example, Milo et al.^23^ reported a low GFR (76.6 mL/min per 1.73 m^2^among regularly transfused patients with β-thalassemia major (TM) and Hamed et al. found impaired eGFR (<90 ML/minute/1.73 m^2^) in 58.82% and 45.71% of patients with and without chelation therapy.^25^Cystatin C has been suggested as a sensitive marker of GFR providing an early indication of renal impairment, possibly superior to serum creatinine.^12^

Shlipak et al. have demonstrated that the addition of cystatin C measurement in calculating the e-GFR significantly improves the risk classification for end stage renal disease.^26^

In this study, we found that the level of serum cystatin C was increased in 33.2% of all patients, but only 2% of patients had abnormal levels of serum creatinine. Other studies such as those performed by Hamed et al. and Economou et al. have also indicated the elevation of serum cystatinC among thalassemic patients.^25^^, ^^27^Economou et al. reported that 36% of their β-TM patients had elevated cystatin C levels.

We found that in 50% of our patients, the urinary NAG level was elevated. The urine of healthy humans contains a small amount of NAG. Increased excretion of NAG in urine has been associated with tubular dysfunction.^28^ There are previously published studies demonstrating the excess secretion of NAG in thalassemic patients which is in agreement with our findings.^7^^,^^18^^,^^21^^,^^25^^,^^29^^, ^^30^

Tantawyet al.^7^ reported elevated NAG levels in 58% of their patients with thalassemia major and intermedia. Mohkamet al.^21^ reported abnormal levels of urinary NAG in 35.9% of patients (confidence interval 26-45%). This percentage was 58% in the study by Ahmadian et al.^30^

The results of our study demonstrated that the urine calcium to creatinine ratio was increased in 160 patients (79.2%). This finding is similar to previously reported results.^10^^, ^^21^^, ^^27^^, ^^31^ Higher transfusion intensity is associated with lower creatinine clearance but more frequent hypercalciuria.^10^

In summary, we found kidney hemosiderosis in a majority of Iranian transfusion-dependent thalassemia major patients. Similar to previous reports in literature, half of our patients had diminished e-GFR and elevation of cystatin C which is in favor of glomerular dysfunction. The results of this study also demonstrated an increase in urinary NAG among 33.2% of our patients and hypercalciuria in 79.2% of them. Both of these findings have been reported in proximal tubulopathy.

## CONCLUSION

 Renal hemosiderosis and asymptomatic renal dysfunction are prevalent among transfusion- dependent β-thalassemia major patients which necessitate regular screening with early markers of glomerular and tubular dysfunction. Further studies in order to investigate the correlation between renal hemosiderosis and early markers of kidney dysfunction among these patients are recommended.
